# Clinicopathological features of adult-onset neuronal intranuclear inclusion disease

**DOI:** 10.1093/brain/aww249

**Published:** 2016-10-25

**Authors:** Jun Sone, Keiko Mori, Tomonori Inagaki, Ryu Katsumata, Shinnosuke Takagi, Satoshi Yokoi, Kunihiko Araki, Toshiyasu Kato, Tomohiko Nakamura, Haruki Koike, Hiroshi Takashima, Akihiro Hashiguchi, Yutaka Kohno, Takashi Kurashige, Masaru Kuriyama, Yoshihisa Takiyama, Mai Tsuchiya, Naoyuki Kitagawa, Michi Kawamoto, Hajime Yoshimura, Yutaka Suto, Hiroyuki Nakayasu, Naoko Uehara, Hiroshi Sugiyama, Makoto Takahashi, Norito Kokubun, Takuya Konno, Masahisa Katsuno, Fumiaki Tanaka, Yasushi Iwasaki, Mari Yoshida, Gen Sobue

**Affiliations:** aww249-1 1 Department of Neurology, Nagoya University Graduate School of Medicine, Nagoya, Aichi, Japan; aww249-2 2 Department of Therapeutics for Intractable Neurological Disorders, Nagoya University Graduate School of Medicine, Nagoya, Aichi, Japan; aww249-3 3 Department of Neurology, Oyamada Memorial Spa Hospital, Yokkaichi, Mie, Japan; aww249-4 4 Department of Neurology and Geriatrics, Kagoshima University Graduate School of Medical and Dental Sciences, Kagoshima, Japan; aww249-5 5 Department of Neurology, Ibaraki Prefectural University of Health Sciences, Ami, Ibaraki, Japan; aww249-6 6 Department of Neurology, National Hospital Organization Kure Medical Centre, Kure, Hiroshima, Japan; aww249-7 7 Department of Neurology, Ota Memorial Hospital, Fukuyama, Hiroshima, Japan; aww249-8 8 Department of Neurology, University of Yamanashi, Kofu, Yamanashi, Japan; aww249-9 9 Department of Neurology, Kosei Chuo General Hospital, Tokyo, Japan; aww249-10 10 Department of Neurology, Kobe City Medical Center General Hospital, Kobe, Hyogo, Japan; aww249-11 11 Department of Neurology, Tottori Prefectural Central Hospital, Tottori, Japan; aww249-12 12 Department of Neurology, National Hospital Organization Utano Hospital, Kyoto, Japan; aww249-13 13 Department of Neurology, Kanto Central Hospital, Tokyo, Japan; aww249-14 14 Department of Neurology, Dokkyo Medical University, Tochigi, Japan; aww249-15 15 Department of Neurology, Nagaoka Red Cross Hospital, Nagaoka, Niigata, Japan; aww249-16 16 Department of Neurology and Stroke Medicine, Yokohama City University Graduate School of Medicine, Yokohama, Kanagawa, Japan; aww249-17 17 Department of Neuropathology, Institute for Medical Sciences of Aging, Aichi Medical University, Nagakute, Aichi, Japan; aww249-18 18 Brain and Mind Research Center, Nagoya University Graduate School of Medicine, Nagoya, Aichi, Japan

**Keywords:** intranuclear inclusion, leukoencephalopathy, dementia, diffusion-weighted image, skin biopsy

## Abstract

Neuronal intranuclear inclusion disease (NIID) is a slowly progressive neurodegenerative disease characterized by eosinophilic hyaline intranuclear inclusions in the central and peripheral nervous system, and also in the visceral organs. NIID has been considered to be a heterogeneous disease because of the highly variable clinical manifestations, and ante-mortem diagnosis has been difficult. However, since we reported the usefulness of skin biopsy for the diagnosis of NIID, the number of NIID diagnoses has increased, in particular adult-onset NIID. In this study, we studied 57 cases of adult-onset NIID and described their clinical and pathological features. We analysed both NIID cases diagnosed by post-mortem dissection and by ante-mortem skin biopsy based on the presence of characteristic eosinophilic, hyaline and ubiquitin-positive intanuclear inclusion: 38 sporadic cases and 19 familial cases, from six families. In the sporadic NIID cases with onset age from 51 to 76, dementia was the most prominent initial symptom (94.7%) as designated ‘dementia dominant group’, followed by miosis, ataxia and unconsciousness. Muscle weakness and sensory disturbance were also observed. It was observed that, in familial NIID cases with onset age less than 40 years, muscle weakness was seen most frequently (100%), as designated ‘limb weakness group’, followed by sensory disturbance, miosis, bladder dysfunction, and dementia. In familial cases with more than 40 years of onset age, dementia was most prominent (100%). Elevated cerebrospinal fluid protein and abnormal nerve conduction were frequently observed in both sporadic and familial NIID cases. Head magnetic resonance imaging showed high intensity signal in corticomedullary junction in diffusion-weighted image in both sporadic and familial NIID cases, a strong clue to the diagnosis. All of the dementia dominant cases presented with this type of leukoencephalopathy on head magnetic resonance imaging. Both sporadic and familial NIID cases presented with a decline in Mini-Mental State Examination and Frontal Assessment Battery scores. Based on these clinicopathological features, we proposed a diagnosis flow chart of adult-onset NIID. Our study suggested that the prevalence rate of adult-onset NIID may be higher than previously thought, and that NIID may be underdiagnosed. We should take NIID into account for differential diagnosis of leukoencephalopathy and neuropathy.

## Teaser

Neuronal intranuclear inclusion disease (NIID) has highly variable clinical manifestations. Sone *et al*. describe the clinical and pathological features of 57 adult-onset cases diagnosed by postmortem dissection/antemortem skin biopsy. They report ‘dementia dominant’ and ‘limb weakness’ subtypes, and recommend consideration of NIID in the differential diagnosis of leukoencephalopathy and neuropathy.

## Introduction

Neuronal intranuclear inclusion disease (NIID), also known as neuronal intranuclear hyaline inclusion disease (NIHID) or intranuclear inclusion body disease (INIBD), is a slowly progressive neurodegenerative disease characterized by eosinophilic hyaline intranuclear inclusions in the central, peripheral and autonomic nervous system cells, and also in visceral organs cells.

The first case of NIID was reported in 1968 (Lindenberg *et al.*, 1968). Since then, many NIID cases have been reported after post-mortem examination (Schuffler *et al.*, 1978; Michaud and Gilbert, 1981; Patel *et al.*, 1985; Munoz-Garcia and Ludwin, 1986; Oyer *et al.*, 1991; Weidenheim and Dickson, 1995; Takahashi-Fujigasaki, 2003; Liu *et al.*, 2008). The onset of disease varies from infancy to the sixties, but two-thirds of them were infantile or juvenile cases (Funata *et al.*, 1990; Zannolli *et al.*, 2002; Takahashi-Fujigasaki, 2003). Information on the clinical manifestations of adult-onset NIID is lacking, and there is no established clinical picture of it yet. A wide range of clinical manifestations has been reported: pyramidal and extrapyramidal symptoms, cerebellar ataxia, dementia, convulsions, neuropathy, and autonomic dysfunction. This wide range of clinical phenotypes makes ante-mortem diagnosis of NIID difficult, especially in adult-onset NIID cases. Until 2011, the number of reported NIID cases was limited to about 40 cases. One of the reasons for this is surely the small chance of diagnosis of NIID, due to it being limited to post-mortem dissection.

Some reports have described an ante-mortem diagnosis of NIID by rectal biopsy or gastrointestinal specimen removed by operation (Schuffler *et al.*, 1978; Goutieres *et al.*, 1990; Barnett *et al.*, 1992; Zannolli *et al.*, 2002; Kulikova-Schupak *et al.*, 2004) and sural nerve biopsy (Sone *et al.*, 2005). Both rectal and sural nerve biopsy cause significant physical stress, limiting their use. Rectal biopsy requires submucosal dissection to pick up myenteric plexus neurons, which carries a risk of perforation (Mizushima *et al.*, 2015). Moreover, one autopsy-diagnosed NIID case reported that rectal biopsy failed to identify intranuclear inclusion ante-mortem (Wiltshire *et al.*, 2010). Endoscopic submucosal dissection, therefore, cannot be used as a screening test for NIID, as for other neurodegenerative disease.

Recently, we reported the usefulness of skin biopsy and detection of intranuclear inclusions exactly identical, both morphologically and immunohistochemically, to those found in the central nervous tissues for the ante-mortem diagnosis of both sporadic NIID cases (Sone *et al.*, 2014) and familial NIID cases (Sone *et al.*, 2011). After these reports, the number of NIID cases that have been diagnosed with skin biopsy has increased (Kitagawa *et al.*, 2014; Sasaki *et al.*, 2015; Toyota *et al.*, 2015). In addition, a characteristic high intensity signal in the corticomedullary junction in diffusion-weighted imaging (DWI) of head MRI has become another clue to NIID diagnosis (Sone *et al.*, 2014), as this MRI image has been seen restricted to the cases with positive skin biopsy with intranuclear inclusions or those with post-mortem histopathological confirmation (Sone *et al.*, 2014). In particular, diagnosis of adult-onset NIID cases with leukoencephalopathy and dementia are increasing, suggesting that the prevalence rate of the adult onset form is higher than we believed.

In this study, we analysed clinicopathological features of 57 cases of adult-onset NIID, both sporadic and familial. Most of these cases were diagnosed with skin biopsy triggered by DWI high intensity signal in corticomedullary junction on head MRI. We characterized clinical manifestations, MRI findings and pathological features of these adult-onset NIID cases, and proposed a diagnosis flow chart of adult-onset NIID.

## Materials and methods

### Cases and clinical assessment

We included the cases as NIID based on the diagnosis by post-mortem dissection, and by ante-mortem skin biopsy with the detection of characteristic intranuclear inclusions identical to those in the CNS tissues. One NIID case was first diagnosed by skin biopsy and confirmed by post-mortem dissection. We performed skin biopsy on the NIID suspected cases with DWI high intensity signal in corticomedullary junction on head MRI, and familial suspected cases with the same clinical features with histopathologically diagnosed NIID case in the same family. The DWI high intensity in the corticomedullary junction in MRI is highly restricted to the cases with intranuclear inclusions in the skin biopsy or post-mortem brain pathology. Thus, this particular DWI high intensity is the possible diagnostic marker of NIID. Intranuclear inclusions in the skin biopsy samples were confirmed on 37 sporadic NIID cases and 15 familial NIID cases from six families studied in this study, including cases we reported previously (Sone *et al.*, 2005; Kitagawa *et al.*, 2014; Sone *et al.*, 2014). We also examined 52 neurologically normal control cases: 16 cases for the histopathological study (between 54 and 84 years of age, average 70.4), and 36 cases for head MRI study (between 49 to 91 years of age, average 66.6).

In this study, we defined familial NIID cases as: at least one NIID patient was diagnosed histopathologically in one family, and the other patient presented with DWI high intensity signal in corticomedullary junction in MRI findings, or medical examination of neurologist confirmed the same phenotype with histopathologically diagnosed NIID patient in the same family. We defined cases that did not meet the above familial NIID definition as sporadic NIID cases, even if the patient had a family history of dementia or neuropathy when these family members were not diagnosed by histopathological evaluation or MRI evaluation.

The detailed clinical data on the patients were obtained from each patient and patient’s families or from clinical records. Neurological examinations were performed repetitively by neurologists. Muscle strength, sensory impairment and autonomic symptoms were assessed. Presence of dementia was assessed by the interviewing of the patient or patient’s family about the presence of forgetfulness or difficulty and mistakes in daily routine tasks. Patients were also screened for executive and cognitive functions using the Mini-Mental State Examination (MMSE) and/or Frontal Assessment Battery (FAB). We used a score of 24 as the MMSE as in previous reports (Folstein *et al.*, 1975; Diniz *et al.*, 2007), and the published age-matched average as the cut-off score for the FAB (Dubois *et al.*, 2000; Appollonio *et al.*, 2005) for impaired cognitive function. Appearance of abnormal behaviour including irritability, cryptic speech, gambling addiction, behavioural apraxia, uninvited behaviour and others were assessed by examining the patient or interviewing patient’s family. Episodes of generalized convulsion, unconsciousness and the appearance of an encephalitic episode such as subacute onset of unconsciousness with fever, headache and vomiting were also assessed by examining the patient or interviewing family. Patients received head MRI, single photon emission computer tomography (SPECT), routine blood chemistry, urinalysis, CSF examination, and nerve conduction studies.

This study was performed with ethical approval of the Ethics Review Committee of Nagoya University School of Medicine. Written informed consent was obtained from all patients.

### Electrophysiological assessment

Motor and sensory conduction was measured in the median, tibial and sural nerves, using a standard method with surface electrodes for stimulation and recording (Kimura, 2001*a*, *b*). Control values of nerve conduction velocity and amplitude were obtained from our previous report (Koike *et al.*, 2003). We defined the slowing conduction velocity when conduction velocity of each case showed below the value of control mean –2 standard deviations (SD). In the same way, we defined compound muscle action potential or sensory nerve action potential reduction as when either compound muscle or sensory nerve action potentials showed below the value of control mean −2 SD.

### Pathological assessment of autopsied cases and skin biopsy

At autopsy, brain, spinal cord and other organs were fixed in 10% formalin solution. Samples of the main representative regions of each organ were embedded in paraffin and sectioned in 6 -μm thickness, and stained with haematoxylin and eosin and Klüver-Barrera. Skin biopsy, and following immunostaining and immunofluorescence staining were performed as described previously (Sone *et al.*, 2011) with anti-ubiquitin antibody (Z0458, Dako) and anti-p62 antibody (sc-28359, Santa Cruz Biotechnology). For assessment of intranuclear inclusion frequency, we prepared a coronal section of cerebral hemisphere, including basal ganglia, stained with anti-p62 antibody using the LSAB Kit (Dako). We selected five random microscopic fields under a ×20 objective lens in each gyrus or basal ganglion, counted the number of neurons, astrocytes and p62-positive intranuclear inclusions and assessed the frequency of intranuclear positive neurons and astrocytes. Samples for electron microscopic study were prepared as described previously (Koike *et al.*, 2007).

### *FMR1* gene analysis

As the case with *FMR1* premutation shows the symptoms and intranuclear inclusions as NIID, we performed *FMR1* gene analysis. Genomic DNA was isolated from peripheral blood leucocytes using standard methods. Southern blotting was performed as described previously (Maddalena *et al.*, 2001). We defined normal alleles as range of 5 to 44 CGG repeats, and premutation alleles as 55 to 230 CGG repeats.

## Results

### Histopathological assessment for the diagnosis of neuronal intranuclear inclusion disease

**Figure 1:**
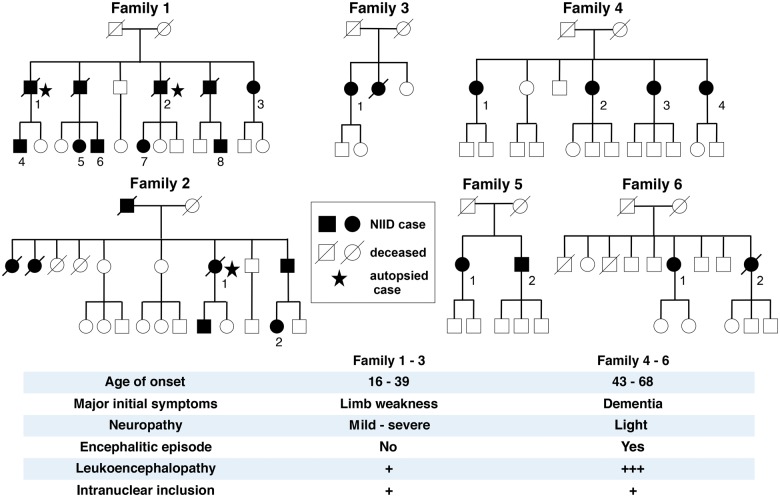
**Family trees and major clinical phenotype of familial NIID**.

We analysed 38 sporadic NIID cases, 19 familial NIID cases from six families and 16 neurologically normal controls (Fig. 1, Tables 1 and 2, and Supplementary Tables 1–3). Of the sporadic NIID cases, Subject S-5 was diagnosed by autopsy, Subject S-31 was diagnosed by both autopsy and skin biopsy, and the others were diagnosed by skin biopsy. Of the familial NIID cases, Family 1 and Family 2 cases were diagnosed by post-mortem dissection and skin biopsy, the other familial cases were diagnosed by skin biopsy.

**Table 1:** Distributions of intranuclear inclusions in autopsied adult onset NIID patients

Histological position, cell			Sporadic cases		Familial cases		
			S-5	S-31	F1-1	F1-2	F2-1
**CNS**	Frontal lobe	N I	+	+	+	+	+
		A I	+	+	+	+	+
	Precentral gyrus	N I	+	+	+	+	+
		A I	+	+	+	+	+
	Temporal lobe	N I	+	+	+	+	+
		A I	+	+	+	+	+
	Occipital lobe	N I	+	+	+	+	+
		A I	+	+	+	+	+
	Hippocampus	N I	+	+	+	+	+
		A I	+	+	+	+	+
	Putamen	N I	+	+	+	+	−
		A I	+	+	+	+	+
	Hypoglossal nucleus	N I	−	−	+	+	+
		A I	+	+	+	+	+
	Cerebellum	P I	+	+	+	+	+
		A I	+	+	+	+	+
	Spinal cord anterior horn	N I	n.a.	+	+	+	+
		A I	n.a.	+	+	+	+
**PNS**	Sympathetic ganglia	N I	n.a.	+	+	+	+
	Dorsal root ganglia	N I	n.a.	+	n.a.	+	+
	Myenteric ganglia	N I	+	+	+	+	+
	Schwann cell inclusion		+	+	+	+	+
**Somatic cells**	Adipocyte (skin)		+	+	+	+	n.a.
	Adrenal mudulla		+	n.a.	+	n.a.	+
	Renal tubule		+	+	+	+	+
	Cadiomyocyte		n.a.	+	+	+	n.a.
	Skeletal muscle cell		−	−	−	−	n.a.
	Smooth muscle cell		+	+	+	+	+
	Hepatocyte		n.a.	−	−	−	n.a.
**Spongiosis of cereberum white matter**			+	+	+	+	+

N I = neuronal intranuclear inclusion; A I = astrocyte intranuclear inclusion; P I = purkinje cell intranuclear inclusion; n.a = not available; + = intranuclear inclusion present; – = intranuclear inclusion absent.

**Table 2:** Summary of clinical features of NIID cases

	Sporadic cases *n = *38	Familial cases		
		Total *n = *19	Total *n = *19	
			Limb weakness (Family 1–3) *n = *11	Dementia (Family 4–6) *n = *8
**Diagnosis**				
Skin biopsy	37 (97.4%)	15 (78.9%)	8 (72.7%)	7 (87.5%)
Autopsy	2 (2.6%)	3 (15.8%)	3 (37.5%)	0 (0%)
**Average onset age (range)**	63.6 (51–76)	39.6 (16–68)	27.5 (16–39)	56.2 (43–68)
**Average disease duration (range)**	5.3 (1–19)	15.4 (1–44)	21.1 (3–44)	7.6 (1–15)
**Sex ratio (male/female)**	13/25	6/13	5/6	1/7
**Clinical manifestations**				
Muscles weakness	10/37 (27%)	18/19 (94.7%)	11/11 (100%)	7/8 (87.5%)
Sensory disturbance	10/35 (28.6%)	13/18 (72.2%)	9/11 (81.8%)	4/7 (57.1%)
Autonomic impairment				
Vomiting	6/38 (15.8%)	6/19 (31.6%)	5/11 (45.5%)	1/8 (12.5%)
Bladder dysfunction	12/36 (33.3%)	10/16 (62.5%)	7/11 (63.6%)	3/5 (60%)
Syncope	3/37 (8.1%)	0/19 (0%)	0/11 (0%)	0/8 (0%)
Miosis	17/18 (94.4%)	7/11 (63.6%)	3/5 (60%)	4/6 (66.7%)
Dementia	36/38 (94.7%)	9/19 (47.4%)	1/11 (9.1%)	8/8 (100%)
Tremor	9/38 (23.7%)	3/19 (15.8%)	0/11 (0%)	3/8 (37.5%)
Rigidity	7/38 (18.4%)	3/18 (16.7%)	0/11 (0%)	3/7 (42.9%)
Ataxia	19/36 (52.8%)	1/18 (5.6%)	0/11 (0%)	1/7 (14.3%)
Abnormal behaviour	10/38 (26.3%)	2/19 (10.5%)	0/11 (0%)	2/8 (25%)
Generalized convulsion	5/38 (13.2%)	1/19 (5.3%)	0/11 (0%)	1/8 (12.5%)
Disturbance of consciousness	15/38 (39.5%)	5/19 (26.3%)	1/11 (9.1%)	4/8 (50%)
Encephalitic episode	8/38 (21%)	1/19 (5.3%)	0/11 (0%)	1/8 (12.5%)
**Head-MRI**				
Leukoencephalopathy	37/38 (97.4%)	10/13 (76.9%)	2/5 (40%)	8/8 (100%)
DWI U-fibre high	38/38 (100%)	9/11 (81.8%)	1/3 (33.3%)	8/8 (100%)
Ventricular distension	37/38 (97.4%)	10/13 (76.9%)	2/5 (40%)	8/8 (100%)
**SPECT**				
Hypoperfusion in cerebral cortex	23/24 (95.8%)	4/5 (80%)	2/2 (100%)	2/3 (66.6%)
**Executive function tests**				
MMSE (<24)	18/33 (54.5%)	0/8 (0%)	0/3 (0%)	0/5 (0%)
FAB (<age matched average)	18/19 (94.7%)	3/3 (100%)	2/2 (100%)	1/1 (100%)
**Laboratory data**				
Serum CK (male: >260 IU/l, female: >170 IU/l)	3/33 (9.1%)	8/14 (57.1%)	7/8 (87.5%)	1/6 (16.6%)
CSF				
Cell (>5/mm^3^)	5/29(17.2%)	0/10 (0%)	0/3 (0%)	0/7 (0%)
Protein (>45 mg/dl)	19/29 (65.5%)	4/10 (40%)	1/3 (33.3%)	3/7 (42.9%)
Glucose (<50mg/dl)	0/29 (0%)	0/8 (0%)	0/3 (0%)	0/5 (0%)
HgbA1c (NGSP) (≥6.2%)	7/33 (21.2%)	2/10 (20%)	2/6 (33.3%)	0/4 (0%)
*FMR1* premutation	0/27 (0%)	0/9 (0%)	0/7 (0%)	0/2 (0%)
**Nerve conduction**				
Motor				
MCV slowing	30/31 (96.7%)	10/12 (83.3%)	8/8 (100%)	2/4 (50%)
CMAP reduction	6/31 (19.4%)	5/11 (45.5%)	5/7 (71.4%)	0/4 (0%)
Sensory				
SCV slowing	23/31 (74.2%)	10/12 (83.3%)	8/8 (100%)	2/4 (50%)
SNAP reduction	4/31 (12.9%)	6/11 (54.5%)	5/7 (71.4%)	1/4 (25%)

Except for diagnosis, average onset age, average disease duration and sex ratio, we described each value as the number of incidence cases / the number of available cases (%). We calculated incidence rates (%) for each value with available case number for each item (Supplementary Tables 1–3). We adopted 24 as the cut-off score for MMSE as in previous reports (Folstein *et al.*, 1975; Diniz *et al.*, 2007), and published age-matched average as cut-off score for FAB score of each case (Dubois *et al.*, 2000; Appollonio *et al.*, 2005). Motor nerve conduction velocity slowing, sensory nerve conduction velocity slowing, compound muscle- and sensory nerve action potential reduction were determined that each value below control average value −2 SD (Koike *et al.* 2003). We treated Subject F3-1 as limb weakness based on their initial symptom. NGSP = national glycohaemoglobin standardization program; CK = creatine kinase; MCV = motor nerve conduction velocity; CMAP = compound muscle action potential; SCV = sensory nerve conduction velocity; SNAP = sensory nerve action potential.

**Figure 2:**
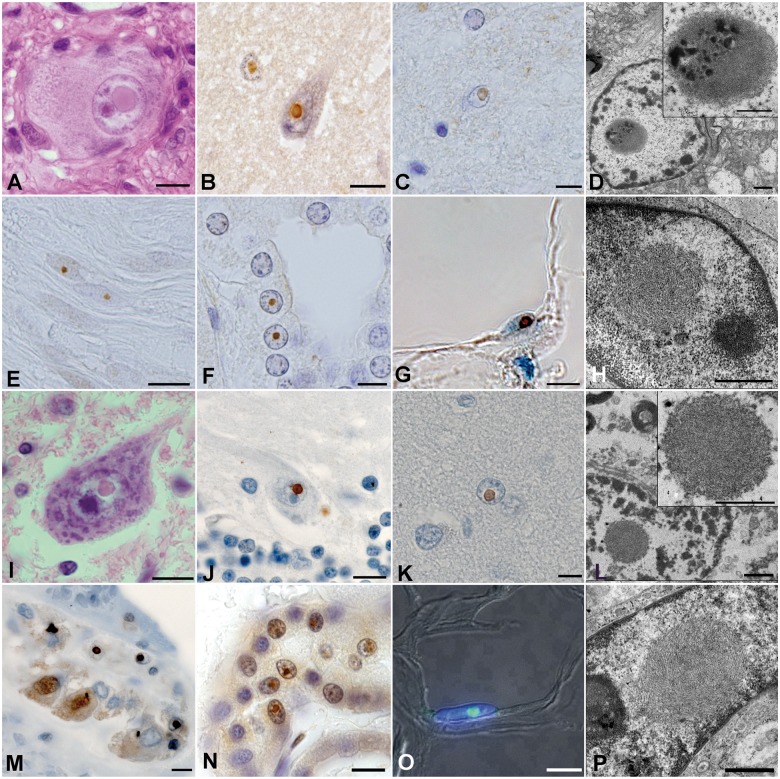
**Histopathological findings of NIID cases.** (**A–H**) Sporadic NIID cases. (**I–P**) Familial NIID cases. (**A** and **I**) Haematoxylin and eosin stain of sympathetic ganglion of Subject S-31(**A**) and spinal cord motor neuron of Subject F2-1 (**I**). (**B**, **C**, **E**, **F**, **J** and **M–O**) Immunostained samples with anti-ubiquitin antibody of temporal cortex neuron of Subject S-31 (**B**), cingulate gyrus astrocyte of Subject S-5 (**C**), dorsal root Schwann cells of Subject S-31 (**E**), renal tubule cells of Subject S-5 (**F**), Perkinje cell of Subject F1-2 (**J**), myenteric plexus neuron of Subject F1-1 (**M**), renal tubule cells of Subject F2-1 (**N**), adipocyte of Subject F3-1 (**O**). (**G** and **K**) Immunostained samples with anti-p62 antibody of adipocyte of Subject S-1 (**G**) and astrocytes of Subject F1-2 (**K**). (**D**, **H**, **L** and **P**) Electron microscopic images of astrocyte of Subject S-31 (**D**), skin fibroblast of Subject S-31 (**H**), astrocyte of Subject F1-2 (**L**), and skin fibroblast of Subject F5-1(**P**). Scale bars in **A–C**, **E–G, I–K** and **M–O** = 10 μm; in **D**, **H**, **L** and **P** = 1 μm.

Histopathological findings of autopsied NIID cases showed that eosinophilic intranuclear inclusions were widely observed in the CNS: in the cerebral cortex, basal ganglia, brainstem and spinal cord, and both in neurons and astrocytic glial cells (Table 1 and Fig. 2). Intranuclear inclusions were also observed in the peripheral nervous system cells and somatic cells of a wide variety of organs except skeletal muscle and hepatocytes (Fig. 2E, F, M, N and Table 1). These inclusions were round-shaped with a diameter of 1.5–10 μm, located in the vicinity of the nucleolus (Fig. 2A–F and I–N). The inclusions were ubiquitin-positive (Fig. 2B, C, E, F, J, M and N), p62-positive (Fig. 2K) and were composed of dense filamentous materials without membrane structure under electron microscopy (Fig. 2D and L).

Skin biopsy samples of NIID cases showed eosinophilic ubiquitin-positive and p62-positive intranuclear inclusions in adipocytes, fibroblasts and sweat gland cells (Fig. 2G and O), round-shaped with diameter of 1.5–3 μm. Electron microscopy imaging showed dense filament material without membrane (Fig. 2H and P). Light and electron microscopic features of these inclusions in the skin were almost identical to those of intranuclear inclusions in the CNS. Furthermore, the light and electron microscopic features of intranuclear inclusions on autopsy and in the skin biopsy samples mentioned above were almost identical between sporadic and familial NIID (Table 1 and Fig. 2). These identical histopathological features among the inclusion in the skin and central nervous tissues form the background of the diagnosis of NIID with skin biopsy findings.

**Figure 3:**
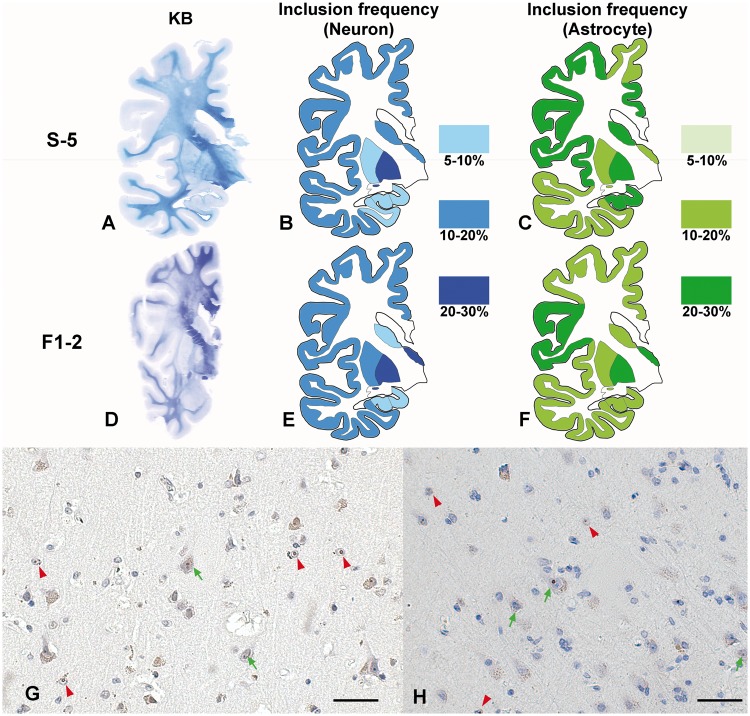
**Frequency of intranuclear inclusion in the NIID brain.** (**A–C**) Sporadic NIID Subject S-5. (**D–F**) Familial NIID case Subject F1-2. (**A** and **D**) Klüver-Barrera stain of coronal section of cerebral hemisphere. White matter of frontal lobe in both samples showed evident demyelination. (**B**, **C**, **E** and **F**) Schematic diagrams of frequency and distribution of intranuclear inclusion of neurons (**B** and **E**) and astrocytes (**C** and **F**) in cerebral cortex, basal ganglia and hippocampus. Frequencies were assessed in serial section of Klüver-Barrera slide with immunohistochemistry with anti-p62 antibody. In Subject F1-2, hippocampus was not included in large coronal section, we analysed frequencies with other hippocampus slide separately. (**G** and **H**) p62 immunostaining of cerebral cortex of frontal lobe of Subjects S-5 (**G**) and F1-2 (**H**). Green arrow shows intranuclear inclusion positive neuron and red arrowhead shows intranuclear inclusion positive astrocyte. Scale bar = 50 μm.

We also investigated the frequency distribution of intranuclear inclusion-positive neurons and astrocytes in the cerebral cortex, hippocampus and basal ganglia of Subject S-5: sporadic NIID case, and Subject F1-2: familial NIID case (Fig. 3). The frequency of intranuclear inclusion in neurons was ∼5–30% in the cerebral cortex and basal ganglia, with a slightly lower frequency in the hippocampus. Frequencies of astrocytes in both cases were similarly between 10% and 30%. Thus, the frequency distribution of intranuclear inclusion of neurons and astrocytes was similar among sporadic and familial NIID cases.

We also studied 16 neurologically normal control samples of the frontal lobe, cerebellum, gastro intestinal tract and kidney, and did not find any intranuclear inclusions in these normal samples.

### Sporadic neuronal intranuclear inclusion disease

#### Clinical manifestations

Details of clinical manifestations of NIID cases are summarized in Table 2 and Supplementary Tables 1 and 2. The onset age of sporadic NIID cases was between 51 and 76. Disease duration ranged from 1 to 19 years. Most sporadic NIID cases presented with dementia as the initial and main clinical manifestation. Dementia was observed in almost all the cases (94.7%). Autonomic impairment of miosis and bladder dysfunction manifesting as urinary incontinence was frequently seen (Table 2 and Supplementary Table 1). Muscle weakness (27%) and sensory disturbance (28.6%) were also observed but not as frequently as in familial cases. Muscle weakness presented with mild extent in the extremities. Sensory disturbance presented with light deterioration of vibratory sense, and sometimes distal dominant numbness in the lower extremities. Tremor, rigidity and ataxia were also observed.

In 26.3% of sporadic NIID cases, patients presented with abnormal behaviour (Table 2 and Supplementary Table 2) such as irritability, cryptic speech, apraxia (e.g. trying to wear shirts as trousers), gambling addiction, disinhibition (such as smoking in non-smoking areas), and some other abnormal behaviours: putting clothes in the refrigerator and complaining ‘I cannot understand everything’, as examples.

Generalized convulsions were observed in 13.2% of sporadic cases. Disturbance of consciousness was often observed (39.5%). The duration of the disturbance varied from a few hours to several days.

**Figure 4:**
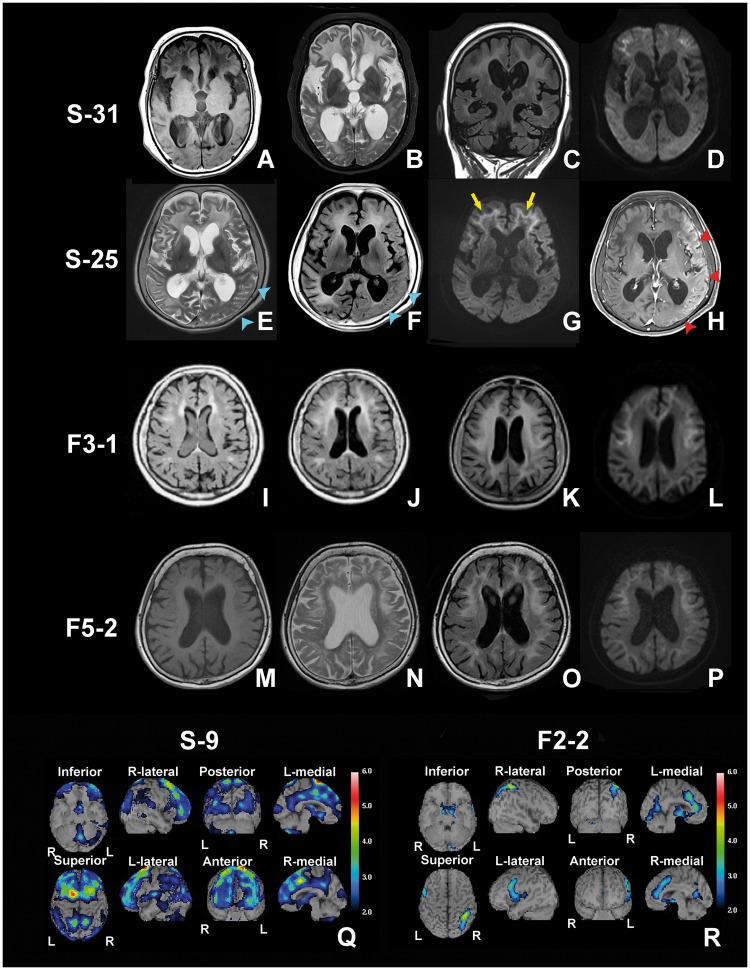
**The results of NIID head MRI and SPECT.** Head MRI findings of Subject S-31 (sporadic NIID) (**A–D**), Subject S-25 (sporadic NIID) (**E–H**), Subject F3-1 (familial NIID) (**I–L**) and Subject F5-2 (familial NIID) (**M–P**). (**A** and **M**) T_1_-weighted image. (**B**, **E** and **N**) T_2_-weighted image. (**C**, **F**, **I–K** and **O**) FLAIR image. (**D**, **G**, **L** and **P**) DWI image. (**H**) Gadolinium-enhanced T_1_-weighted image. (**A–D**) Head MRI findings of Subject S-31 at age 72 years, almost akinetic mutism state. Prominent leukoencephalopathy, DWI high intensity signal in corticomedurally junction and ventricular distention were observed (**D**). (**E–H**) Head MRI findings at the presence of encephalitic symptom of Subject S-25. On T_2_ (**E**) and FLAIR (**F**) image, brain oedema is observed (blue arrowhead). On DWI, oedematous portion showed slightly high intensity but weaker than corticomedurally junction high intensity signal (yellow arrow) (**G**). Oedematous portion showed high intensity on gadolinium enhanced T_1_ image (red arrow head) (**H**). (**I–L**) Progress of leukoencephalopathy in familial NIID Subject F3-1 at age 53 (**I**), age 58 (**J**) and age 64 (**K** and **L**). High intensity area of FLAIR image extended remarkably. (**M–P**) Head MRI findings of familial NIID Subject F5-2 presented dementia dominantly. Findings are almost identical to sporadic case. (**Q** and **R**) The results of easy Z-score imaging system (eZIS) analysed data of ^99m^Tc-ECD SPECT. Subject S-9 showed severe regional cerebral blood flow decline and Subject F2-2 eZIS data showed light regional cerebral blood flow decline.

In 21% of sporadic NIID cases, patients presented with characteristic symptoms of subacute onset encephalitic episodes: fever, headache, vomiting and disturbance of consciousness. These NIID cases with encephalitic episodes were admitted to the hospital via emergency visit and had a normal titre of antibody against viruses. No pleocytosis of CSF ruled out bacterial or viral meningitis and encephalitis (Table 2 and Supplementary Table 3). Some of the cases with an encephalitic episode presented with focal brain oedema with gadolinium enhancement (Fig. 4). High dose steroid administration (1000 mg prednisolone per day intravenously for 3 days) often improved encephalitic symptoms and diminished the brain oedema and gadolinium enhancement, while executive function deteriorated slowly and a state of akinetic mutism often developed in some cases.

#### Laboratory data

Laboratory data are shown in Table 2 and Supplementary Table 3. Three cases (9.1%) of sporadic NIID cases showed elevated serum creatine kinase levels. Elevation of CSF protein level was frequently observed (65.5%). Approximately 20% of cases showed elevated glycated haemoglobin (HgbA1c). Almost all sporadic cases, except Subject S-16, presented abnormal findings of motor or sensory nerve conduction to a variable extent. Motor nerve conduction velocity slowing was most frequently observed in the median and tibial nerves. The distribution of peripheral nerve damage was polyneuropathy type, being distally predominant in four extremities.

#### Neuroradiological findings

Findings of head MRI and SPECT are shown in Table 2, Fig. 4 and Supplementary Table 3.

Head MRI findings, showed obvious leukoencephalopathy in T_2_ and fluid attenuated inversion recovery (FLAIR) image (Fig. 4A–H). A high intensity area was widely observed from the corticomedullary junction to around the root of gyrus. These white matter abnormalities were confluent and bilaterally symmetrical in T_2_ image, but in FLAIR image, the edge of the high intensity area appeared rather dimly and generally prominent in the frontal lobe. It was also observed in the external capsule. The area that showed quite high intensity in the T_2_ image presented a low intensity signal in the T_1_ image (Fig. 4A).

On DWI, high intensity signal along the corticomedullary junction was observed in all sporadic NIID cases (Fig. 4D and G), and spread along the corticomedullary junction with advancement of the disease. At the early stage, the DWI high intensity signal was slightly observed in a small regional portion of the corticomedullary junction in the frontal lobe. As the disease worsened, the DWI high intensity signal extended only along the corticomedullary junction of the cerebrum but did not expand into deep white matter even in the extremely advanced stage of akinetic mutism showing widely expanded leukoencephalopathy in MRI T_2_ image (Fig. 4A–D). Such a DWI high intensity signal mentioned above was not observed in the 36 neurologically normal cases.

It is noteworthy that Subject S-25 showed focal brain oedema on head MRI simultaneously with the emergence of encephalitic symptoms (Fig. 4E–H). These brain oedemas were focal and not symmetrical in T_2_ and FLAIR. The oedematous portion of the brain was not high intensity on DWI (Fig. 4G), and was enhanced by gadolinium (Fig. 4H).

As for SPECT findings, Subject S-9 showed decreases in regional cerebral blood flow (Fig. 4Q). Most of the sporadic NIID cases showed this regionally decreased cerebral blood flow on SPECT (Table 2).

#### *FMR1* CGG repeat analysis

We analysed the CGG repeat length of the *FMR1* gene of cases, with written consent, to exclude fragile X-associated tremor/ataxia syndrome (FXTAS). All cases with analysed *FMR1* showed a normal range of CGG repeats (<44 repeats) (Table 2 and Supplementary Table 3).

#### Executive function tests

Results of MMSE and FAB are summarized in Table 2 and Supplementary Table 2. Almost half of the cases showed decreased MMSE below cut-off score 24 (Folstein *et al.*, 1975; Diniz *et al.*, 2007), and >90% of cases were impaired in FAB scores of published age-matched average (Dubois *et al.*, 2000; Appollonio *et al.*, 2005). The decline of FAB scores was more prominent than those of MMSE scores. Subject S-9 showed marked definitive decline of both FAB and MMSE scores with extensive decline of regional cerebral blood flow in SPECT.

#### Case reports of sporadic NIID

##### Subject S-7

Subject S-7 consulted a neurologist due to forgetfulness reported by family members. She was aged 69 and had no family history or past history of neurological or psychological disease. Neurological examination revealed miosis, with a pupil size of 2 mm/2 mm and prompt light reflex. She did not present weakness on the manual muscles testing nor sensory disturbance, tremor, rigidity, ataxia or urinary incontinence. Tendon reflex showed diffuse hyporeflexia. Executive function was declined (MMSE 19/30 and FAB 6/18). Head MRI T_2_ image showed symmetrical, confluent and frontal dominant leukoencephalopathy. DWI showed high intensity signal along the corticomedullary junction. Skin biopsy revealed abundant intranuclear inclusions.

##### Subject S-9

Subject S-9 presented with a subacute progressive encephalitic episode with fever (38°C), headache, disorientation, dyscalculia and disturbance of consciousness at age 55. He had no family history or past history of neurological or psychological disease. Head MRI showed symmetrical, confluent leukoencephalopathy and DWI showed high intensity signal along the corticomedullary junction. Repeated CSF tests showed no pleocytosis with only mild protein elevation, therefore intracranial infection was excluded. Fever and disturbance of consciousness lasted for 3 weeks and deteriorated, with no evident infectious focus in the whole body and no response to antibiotics therapy. Steroid pulse therapy (intravenous injection of methylprednisolone 1000 mg/day for 3 days) resulted in improved consciousness. MMSE score was 20 at the time of testing, but deteriorated slowly thereafter. Intranuclear inclusions were demonstrated by skin biopsy.

### Familial NIID

#### Clinical manifestations

##### Families 1–3

The onset age of Families 1–3 was between 16 and 39, and disease duration was from 3 to 44 years (Fig. 1, Table 2 and Supplementary Table 1). Main and initial clinical manifestations were limb weakness (100%), sensory disturbance and autonomic symptoms like urinary incontinence. Deterioration was slow. Over 20 years after onset, clinical symptoms became serious (Sone *et al.*, 2005). Subject F3-1, onset age of 39, presented with dementia and leukoencephalopathy at the age of 58, but other cases did not report dementia. Diffuse limb muscle weakness was seen in all cases (Table 2), and distally dominant sensory disturbance (81.8%), autonomic symptoms of vomiting, bladder dysfunction and miosis were frequently observed. While dementia was rarely seen, ataxia, abnormal behaviour, convulsion and encephalitic episode were not seen (Table 2 and Supplementary Table 1). In laboratory data, serum creatine kinase was elevated in 87.5% of examined cases. Motor and sensory nerve conduction abnormality was present in most cases (Table 2 and Supplementary Table 2).

##### Families 4–6

The onset age was between 43 and 68, and disease duration was from 1 to 15 years. The main and initial clinical manifestation was dementia (100%). Cases of these three families did not complain of weakness as the chief and initial symptom but it became evident on medical examination by the neurologist. Autonomic impairment of vomiting, bladder dysfunction and miosis were observed in similar extent to those of Families 1–3 (Table 2 and Supplementary Table 1). Tremor, rigidity, ataxia, abnormal behaviour, disturbance of consciousness and encephalitic episode (Subject F5-1) were similar to the sporadic cases (Table 2 and Supplementary Table 1). In laboratory data, serum creatine kinase elevation was only seen in one of six cases (16.6%). Motor and sensory conduction impairment was mild but present in half of the patients.

#### Neuroradiological findings

##### Families 1 and 2

Among familial NIID cases of Families 1 and 2, head MRI showed in one case of Family 1 (Subject F1-1), with disease duration of 37 years, a mild leukoencephalopathy around the frontal and occipital horn of the lateral ventricle, and ventricular distention (Supplementary Table 2). DWI was not evaluated in Subject F1-1. Other NIID cases of Families 1 and 2 with duration of less than 30 years did not show leukoencephalopathy or ventricular distention. On SPECT finding, familial NIID Subject F2-2 showed mild decreases in regional cerebral blood flow (Fig. 4R).

##### Families 3–6

Head MRI of familial NIID cases in the Families 3–6, showed obvious leukoencephalopathy in T_2_ and FLAIR image in all examined cases (Fig. 4I–P and Supplementary Table 2). DWI high intensity signal along the corticomedullary junction was also observed (Fig. 4L and P) in all the cases. The high intensity area of FLAIR image extended as the disease progressed (Fig. 4I–K). Subject F5-1 showed focal brain oedema on head MRI simultaneously with the emergence of encephalitic symptoms, like some sporadic NIID cases.

#### *FMR1* CGG repeat analysis

Some NIID cases with written consent, in Families 1–5, had *FMR1* analysed, which showed a normal range of the number of CGG repeats (<44 repeats) (Table 2 and Supplementary Table 3).

#### Executive function tests

Familial NIID cases presented with a slight decline of MMSE scores but were above the cut-off score of 24. While FAB scores were almost equal, or slightly below the scores of published age-matched averages (Dubois *et al.*, 2000; Appollonio *et al.*, 2005; Diniz *et al.*, 2007) (Supplementary Table 2), predominantly in cases with age of onset over 40 years of age.

#### Case reports of familial NIID

##### Subject F3-1

Difficulty in jumping was the first symptom noticed by this case, at age 39. She deteriorated slowly. Her younger sister presented with distal dominant muscle weakness and hyporeflexia. At age 49, she presented with mild muscle weakness, hypotonia and hyporeflexia diffusely distributed in all four extremities. Nerve conduction studies showed slowing of motor nerve conduction velocity. At age 53, she complained of dysarthria, and head MRI showed a slight high intensity area around the anterior horn of the lateral ventricle resembling chronic ischaemic change (Fig. 4I), and at age 58, the high intensity area of head MRI extended a little (Fig. 4J). At age 60, 21 years after disease onset, walking became difficult and the patient required a wheelchair. At age 64, head MRI showed evident leukoencephalopathy (Fig. 4K) and DWI high intensity signal in the corticomedullary junction (Fig. 4L). Forgetfulness and defect of working memory while cooking became evident and MMSE was assessed. Decline in the score (25/30) was observed (Supplementary Table 2). Skin biopsy showed intranuclear inclusions.

##### Subject F5-2

Subject F5-2 presented with complaints of forgetfulness, dysarthria, gait disturbance and urinary incontinence at age 54. She sometimes reported dysaesthesia in the face and upper extremities. Screening head MRI showed leukoencephalopathy and NCV showed sensory-motor polyneuropathy. Her younger brother presented with irritability and leukoencephalopathy, and was diagnosed with NIID by skin biopsy. At age 59, neurological examination revealed mild dysarthria, hyporeflexia, and a mild disturbance in pain and temperature sensation in the lower extremities. She could not perform tandem walking nor stand on one leg. FAB score showed definitive decline (14/18), but MMSE score was almost normal (28/30) (Supplementary Table 2). Head MRI showed ventricular distention, leukoencephalopathy in the T_2_ image and high intensity signal along the corticomedullary junction in DWI (Fig. 4M–P).

### Clinical phenotype subgroups with dementia-dominancy and limb weakness-dominancy

**Figure 5:**
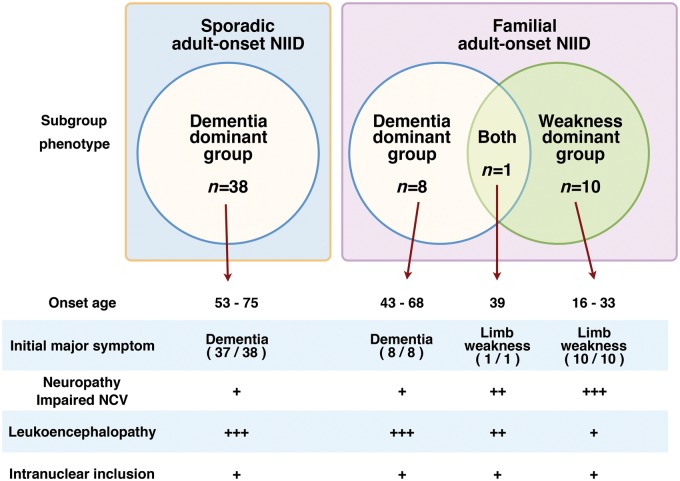
**Sporadic and familial NIID and phenotypic subgroup**.

As a consequence of this study, adult-onset NIID cases can be subgrouped into dementia-dominant and limb weakness-dominant phenotypes (Fig. 5).

The dementia-dominant NIID group had an onset age over 40, and in most cases, dementia was the first symptom and was the reason for consulting a neurologist. Some cases also presented with mild autonomic dysfunction, such as bladder dysfunction and miosis, ataxia, tremor and rigidity (Table 2 and Supplementary Table 1). Most of them did not complain of weakness and sensory disturbance, but subclinical neuropathy was detected by neurological examinations and nerve conduction study, we thus suppose that subclinical neuropathy related to NIID is present.

The limb weakness-dominant group was relatively young at age of onset, 16 to 39 years, and weakness of the lower limbs was the first symptom. This group consists of familial cases with the onset age less than 40 years. As the patients became older, limb weakness worsened slowly and sensory disturbances and autonomic dysfunction developed. Dementia and leukoencephalopathy was relatively mild and did not present before 20 years of disease duration. In some cases of this group, dementia did not present throughout disease duration. However, MMSE and FAB revealed mild decline of these scores, and sponsiosis of white matter was observed in autopsied cerebrum specimens (Fig. 3D). Ataxia, tremor or rigidity was not observed.

Familial NIID Subject F3-1 is of interest, because this patient presented with both clinical manifestations of the weakness-dominant and dementia-dominant phenotypes. This patient presented with limb weakness at age 39, and 20 years after disease onset, at age 58, presented with dementia and obvious leukoencephalopathy (Fig. 4I–L). This case is an example that NIID can present with both limb weakness with polyneuropathy and dementia in one patient with a variable extent of these dominancy.

## Discussion

### Diagnosis of adult-onset neuronal intranuclear inclusion disease

The diagnosis of NIID was made histopathologically by autopsy in five cases and by skin biopsy in the other cases. Histopathological features of intranuclear inclusions of skin biopsy and post-mortem CNS tissue were very similar. The distribution of ubiquitin-positive intranuclear inclusions in all five autopsied cases was almost identical, observed in both neurons and astrocytes, and widely in CNS, peripheral nervous systems and somatic cells, including the skin. In particular, Subject S-31 was diagnosed as NIID by skin biopsy ante-mortem and then confirmed by post-mortem histopathological examination. This case (Subject S-31) is the first NIID case in which eosinophilic ubiquitin-positive intranuclear inclusion was confirmed in both skin biopsy specimen and autopsy examination and presented with leukoencephalopathy and DWI high intensity signal in the corticomedullary junction. This case again confirmed the view of diagnostic usefulness and dependability of skin biopsy (Sone *et al.*, 2011, 2014). Based on these observations, we thought that the presence of characteristic intranuclear inclusions in the biopsied skin could be used as the major diagnostic criteria for NIID.

The light microscopic features of intranuclear inclusion of NIID somewhat resemble Marinesco bodies, which are also stained by eosin, anti-ubiquitin antibody and anti-p62 antibody (Dickson *et al.*, 1990; Kuusisto *et al.*, 2008; Odagiri *et al.*, 2012; Grigoriev *et al.*, 2015). The electron microscopic findings of Marinesco bodies, however, show aggregation of finely granular materials and a filamentous lattice-like structure (Leestma and Andrews, 1969; Okamoto and Hirai, 1981), which is different to those of NIID cases in this study and previous reports (Lindenberg *et al.*, 1968; Takahashi-Fujigasaki, 2003). We therefore consider that the nature of Marinesco bodies differs from intranuclear inclusion of NIID as previously reported (Janota, 1979; Sung, 1980; Oyer *et al.*, 1991).

The histopathological features of NIID partially resemble those of FXTAS. Some cases of FXTAS present with dementia and leukoencephalopathy (Kasuga *et al.*, 2011), and eosinophilic ubiquitin-positive intranuclear inclusions are observed in FXTAS in neurons, glial cells and somatic cells, similar to NIID (Gokden *et al.*, 2009; Hagerman, 2013; Buijsen *et al.*, 2014). There is no literature on the skin pathological or electron microscopic findings of intranuclear inclusions in FXTAS. Therefore, at present, it is difficult to distinguish NIID from FXTAS with only the histopathological findings. Clinical features such as psychiatric problems like depression and anxiety, family history of Fragile X syndrome, and genetic evaluation of *FMR1* premutation are important for the differential diagnosis of NIID and FXTAS. For this reason, we performed gene analysis for FXTAS, and confirmed that this gene mutation was not present in our case series.

### Clinicopathological continuity among dementia-dominant and limb weakness-dominant subgroups

Until now, NIID has been described as a heterogeneous disease entity because of the variety of clinical features and pathological findings (Munoz-Garcia and Ludwin, 1986; Takahashi-Fujigasaki, 2003) with little information about adult-onset NIID cases.

In this study, we described adult-onset NIID cases and divided them into two sub-phenotype groups of dementia-dominant and limb weakness-dominant, based on the early phase of clinical phenotypes. We consider, however, that these two NIID subgroups are not two separate disease entities, but belong to one large disease entity that presents with both dementia with leukoencephalopathy, and limb weakness with polyneuropathy. The clinical manifestations of both groups overlapped (Fig. 5), and pathological features of intranuclear inclusions such as the distribution, immunoreactivity, frequency, white matter damage in Klüver-Barrera stain and electron microscopic findings are identical.

Furthermore, we reported that, in this study, tremor, rigidity and general convulsions were rarely observed in adult-onset NIID (Table 2), and these manifestations were frequently reported in infantile and juvenile NIID cases (Sung *et al.*, 1980; Haltia *et al.*, 1984; Funata *et al.*, 1990; Sloane *et al.*, 1994; O'Sullivan *et al.*, 2000; Paviour *et al.*, 2005). Clinical features of infant, juvenile and adult NIID are different, but some clinical manifestations are common to them. Such a variety of NIID clinical manifestations depending on disease onset ages may resemble Alexander disease that is a white matter disease caused by *GFAP* (glial fibrillary acidic protein) gene mutation and clinical phenotype is different by onset age (Sawaishi, 2009; Prust *et al.*, 2011).

We need to identify the genetic background of NIID, particularly of familial cases, to solve and confirm the clinical entity of NIID. However, in view of the above, we consider that NIID may be one large disease entity with the same histopathological background, and subdivided it into some groups by clinical phenotypes. Based on our results on adult-onset NIID, it is necessary to study the head MRI DWI and skin biopsy of infantile and juvenile NIID cases to extend our understanding of NIID.

### Characteristic symptoms and signs in adult-onset neuronal intranuclear inclusion disease

#### Head MRI findings

Head MRI examination detected remarkable leukoencephalopathy and DWI high intensity signal in the corticomedullary junction in the dementia-dominant group. The DWI high intensity signal is a characteristic finding of NIID patients (Kitagawa *et al.*, 2014; Sone *et al.*, 2014; Sasaki *et al.*, 2015; Toyota *et al.*, 2015) and we could not find such findings in 36 normal controls. In this study, we found that the DWI high intensity signal extended along the corticomedullary junction, but did not expand into the deep white matter, even in the advanced stage. This finding may be a strong clue to the diagnosis of NIID, and we performed skin biopsy on the NIID-suspected patients with these DWI high intensity signals.

#### Subacute encephalitic episode

Nine cases of the dementia-dominant group presented with a subacute encephalitic episode and some of these cases presented with focal brain oedema simultaneously with gadolinium enhancement. However, subacute encephalitic episodes have not been reported as a NIID symptom. Martin and Banker (1969) reported a case that resembled subacute leukoencephalopathy with intranuclear inclusion. Clinical manifestations and features of intranuclear inclusions of this case were fairly similar to our NIID cases. Langford (1994) also reported on a patient, age 38, who presented with headache, progressive weakness, and two brain lesions with oedema and MRI enhancement. A brain biopsy sample revealed selective loss of myelin, reactive astrocytes and glial intranuclear inclusions looking like those of NIID under electron microscopy (Langford, 1994). The authors of both case reports discussed the similarity of clinical and histopathological features of these cases to those of NIID. There are, however, no data on DWI and ubiquitin immunohistochemistry. We consider that these two cases were NIID because of the similarity of intranuclear inclusion and distribution, and thus such an encephalitic episode is one of the NIID manifestations.

To treat these encephalitic episodes, steroid pulse therapy may be effective to some extent to reduce brain oedema and improve consciousness in the short term. Long-term efficacy of steroid pulse therapy is unknown because the number of steroid treated NIID cases is still limited.

#### Impaired cognitive function

In neurobehavioural evaluations, FAB scores more obviously declined than MMSE scores, particularly in the dementia-dominant group. MMSE is useful in the assessment of cortical function, such as language, and relatively insensitive to the executive dysfunction of white matter dementia (Filley, 2012). FAB has been found to be sensitive to white matter dysfunction (Kanno *et al.*, 2011). White matter dementia is distinguished from cortical dementia, like Alzheimer’s disease, by relative normalcy of language function and declarative memory encoding while cognitive speed, executive function, and sustained attention are impaired (Filley, 1998; Prins *et al.*, 2005). The FAB score decline of NIID cases may reflect the white matter damage, and this pattern of decline is similar to other neurological diseases with white matter damage, such as cerebral autosomal dominant arteriopathy with subcortical infarcts and leukoencephalopathy (CADASIL) and FXTAS (Filley *et al.*, 1999; Schmahmann *et al.*, 2008; Chabriat *et al.*, 2009; Kasuga *et al.*, 2011).

### Neuronal intranuclear inclusion disease diagnostic flowchart

**Figure 6:**
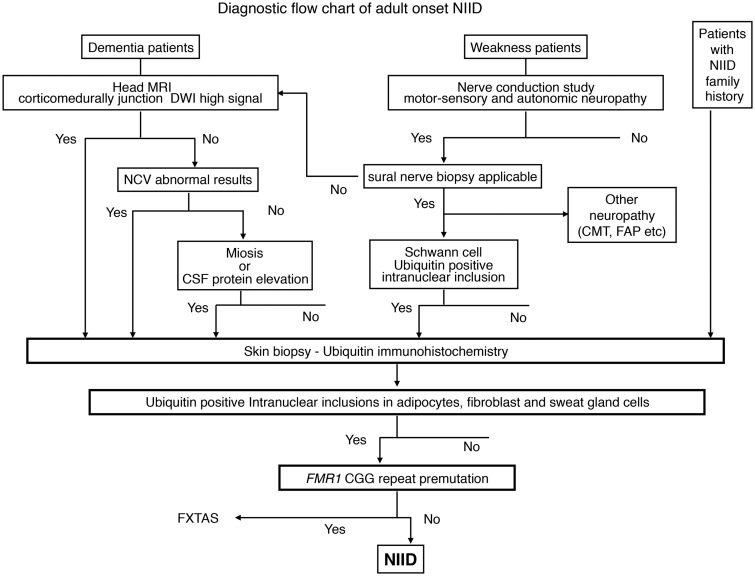
**Proposed flowchart of adult-onset NIID diagnosis**. DWI = diffusion-weighted imaging; FAP = familial amyloid polyneuropathy; NCV = nerve conduction velocity.

As the outcome of this study, we propose the diagnostic flow chart of adult-onset NIID (Fig. 6). In dementia-dominant patients, most of the NIID cases present with leukoencephalopathy and symptoms related to white matter damage. Therefore, the nature of dementia may be different from cortical dementia such as Alzheimer’s disease and subcortical dementia such as Huntington’s disease (Filley, 2012). Some NIID cases did not present with leukoencephalopathy in MRI T_2_ image in our study, and we selected the high intensity signal of corticomedullary junction in the DWI as the strongest and easiest indicator of NIID, regardless of the nature of dementia. Investigation of skin biopsy sample is recommended when DWI high signal of corticomedullary junction is observed.

In case of DWI high negative, the nerve conduction study should be assessed because over 90% of NIID cases present with subclinical neuropathy. Among cases without either DWI high or nerve conduction study abnormal results, miosis or protein elevation of CSF may suggest NIID, because over 50% of NIID cases present these findings, and skin biopsy is worth consideration.

In weakness-dominant patients, nerve conduction must be studied and motor and/or sensory nerve damage is definitive. Suspect NIID if manifestations of autonomic dysfunction, such as miosis or bladder dysfunction are observed. In case of severe sensory nerve damage applicable to sural nerve biopsy, nerve biopsy should be examined, and other neuropathies, such as Charcot-Marie-Tooth disease and familial amyloid polyneuropathy, must be ruled out. On the other hand, in case sural nerve biopsy is impracticable, proceed to the evaluation of head MRI DWI image in the dementia-dominant group flow chart and follow from there.

Skin biopsy and immunohistochemistry procedures are described previously (Sone *et al.*, 2011). We propose that the favourable region of skin biopsy for NIID is 10 cm above lateral malleolus, because of the appropriate density of sweat glands and adipocytes, to evaluate intranuclear inclusions of adipocytes, fibroblasts and sweat gland cells in one biopsy specimen.

In cases with ubiquitin-positive intranuclear inclusions in adipocytes, fibroblasts and sweat gland cells, we suggest genetic testing for *FMR1* CGG repeat expansion to rule out FXTAS. Cases without *FMR1* CGG premutation are finally diagnosed NIID ante-mortem.

In this study, dementia and DWI high signal triggered skin biopsy for most of the NIID cases. However, considering the systemic-wide distribution of intranuclear inclusion and neuronal loss in reported NIID cases (Lindenberg *et al.*, 1968; Patel *et al.*, 1985; Funata *et al.*, 1990; Takahashi-Fujigasaki, 2003; Woulfe, 2007; Liu *et al.*, 2008), we should be aware of the possibility of another phenotype of NIID (neither dementia nor weakness) and accumulate histopathologically-diagnosed NIID cases to refine the whole NIID clinical picture.

## Conclusion

Here, we report on a large number of adult-onset NIID cases and describe its clinicopathological picture for the first time. The morbidity rate of adult-onset NIID may be larger than previously thought, as many cases may remain undiagnosed. We must consider NIID as a differential diagnosis of leukoencephalopathy and neuropathy, using the NIID diagnostic flowchart to gather adult-onset NIID cases and promote research to identify a causative gene.

## Supplementary Material

Supplementary Data

## Abbreviations

DWI: diffusion weighted image
FAB: frontal assessment battery
FLAIR: fluid attenuated inversion recovery
FXTAS: fragile X-associated tremor/ataxia syndrome
MMSE: Mini-Mental State Examination
NIID: neuronal intranuclear inclusion disease
SPECT: single photon emission computer tomography

## References

[aww249-B1] Appollonio I Leone M Isella V Piamarta F Consoli T Villa ML The Frontal Assessment Battery (FAB): normative values in an Italian population sample. Neurol Sci 2005; 26: 108–16. 15995827 10.1007/s10072-005-0443-4

[aww249-B2] Barnett JL McDonnell WM Appelman HD Dobbins WO Familial visceral neuropathy with neuronal intranuclear inclusions: diagnosis by rectal biopsy. Gastroenterology 1992; 102: 684–91. 1310083 10.1016/0016-5085(92)90121-e

[aww249-B3] Buijsen RA Sellier C Severijnen LA Oulad-Abdelghani M Verhagen RF Berman RF FMRpolyG-positive inclusions in CNS and non-CNS organs of a fragile X premutation carrier with fragile X-associated tremor/ataxia syndrome. Acta Neuropathol Commun 2014; 2: 162. 25471011 10.1186/s40478-014-0162-2 PMC4254384

[aww249-B4] Chabriat H Joutel A Dichgans M Tournier-Lasserve E Bousser MG Cadasil. Lancet Neurol 2009; 8: 643–53. 19539236 10.1016/S1474-4422(09)70127-9

[aww249-B5] Dickson DW Wertkin A Kress Y Ksiezak-Reding H Yen SH Ubiquitin immunoreactive structures in normal human brains. Distribution and developmental aspects. Lab Invest 1990; 63: 87–99. 2165197

[aww249-B6] Diniz BS Yassuda MS Nunes PV Radanovic M Forlenza OV Mini-mental State Examination performance in mild cognitive impairment subtypes. Int Psychogeriatr 2007; 19: 647–56. 17502007 10.1017/S104161020700542X

[aww249-B7] Dubois B Slachevsky A Litvan I Pillon B The FAB: a Frontal Assessment Battery at bedside. Neurology 2000; 55: 1621–6. 11113214 10.1212/wnl.55.11.1621

[aww249-B8] Filley CM The behavioral neurology of cerebral white matter. Neurology 1998; 50: 1535–40. 9633691 10.1212/wnl.50.6.1535

[aww249-B9] Filley CM White matter dementia. Ther Adv Neurol Disord 2012; 5: 267–77. 22973423 10.1177/1756285612454323 PMC3437529

[aww249-B10] Filley CM Thompson LL Sze CI Simon JA Paskavitz JF Kleinschmidt-DeMasters BK White matter dementia in CADASIL. J Neurol Sci 1999; 163: 163–7. 10371078 10.1016/s0022-510x(99)00038-6

[aww249-B11] Folstein MF Folstein SE McHugh PR “Mini-mental state”. A practical method for grading the cognitive state of patients for the clinician. J Psychiatr Res 1975; 12: 189–98. 1202204 10.1016/0022-3956(75)90026-6

[aww249-B12] Funata N Maeda Y Koike M Yano Y Kaseda M Muro T Neuronal intranuclear hyaline inclusion disease: report of a case and review of the literature. Clin Neuropathol 1990; 9: 89–96. 1692776

[aww249-B13] Gokden M Al-Hinti JT Harik SI Peripheral nervous system pathology in fragile X tremor/ataxia syndrome (FXTAS). Neuropathology 2009; 29: 280–4. 18627480 10.1111/j.1440-1789.2008.00948.x

[aww249-B14] Goutieres F Mikol J Aicardi J Neuronal intranuclear inclusion disease in a child: diagnosis by rectal biopsy. Ann Neurol 1990; 27: 103–6. 2405768 10.1002/ana.410270117

[aww249-B15] Grigoriev IP Korzhevskii DE Sukhorukova EG Gusel'nikova VV Kirik OV Intranuclear Ubiquitin-Immunopositive Structures of the Human Substantia Nigra Neurons [in Russian]. Tsitologiia 2015; 57: 780–7. 27012092

[aww249-B16] Hagerman P Fragile X-associated tremor/ataxia syndrome (FXTAS): pathology and mechanisms. Acta Neuropathol 2013; 126: 1–19. 23793382 10.1007/s00401-013-1138-1 PMC3904666

[aww249-B17] Haltia M Somer H Palo J Johnson WG Neuronal intranuclear inclusion disease in identical twins. Ann Neurol 1984; 15: 316–21. 6331275 10.1002/ana.410150403

[aww249-B18] Janota I Widespread intranuclear neuronal corpuscles (Marinesco bodies) associated with a familial spinal degeneration with cranial and peripheral nerve involvement. Neuropathol Appl Neurobiol 1979; 5: 311–7. 225694 10.1111/j.1365-2990.1979.tb00630.x

[aww249-B19] Kanno S Abe N Saito M Takagi M Nishio Y Hayashi A White matter involvement in idiopathic normal pressure hydrocephalus: a voxel-based diffusion tensor imaging study. J Neurol 2011; 258: 1949–57. 21512742 10.1007/s00415-011-6038-5

[aww249-B20] Kasuga K Ikeuchi T Arakawa K Yajima R Tokutake T Nishizawa M A patient with fragile x-associated tremor/ataxia syndrome presenting with executive cognitive deficits and cerebral white matter lesions. Case Rep Neurol 2011; 3: 118–23. 21720528 10.1159/000328838 PMC3124446

[aww249-B21] Kimura J Assessment of individual nerves. In: Kumar J, editor. Electrodiagnosis in disease of nerve and muscle: principles and practice. 3rd edn NewYork: Oxford University Press; 2001a, p. 130–77.

[aww249-B22] Kimura J Principles and variations of nerve conduction studies. In: Kimura J, editor. Electrodiagnosis in disease of nerve and muscle: principles and practice3rd edn New York: Oxford University Press; 2001b, p. 91–129.

[aww249-B23] Kitagawa N Sone J Sobue G Kuroda M Sakurai M Neuronal intranuclear inclusion disease presenting with resting tremor. Case Rep Neurol 2014; 6: 176–80. 24987362 10.1159/000363687 PMC4067726

[aww249-B24] Koike H Iijima M Mori K Yamamoto M Hattori N Katsuno M Nonmyelinating Schwann cell involvement with well-preserved unmyelinated axons in Charcot-Marie-Tooth disease type 1A. J Neuropathol Exp Neurol 2007; 66: 1027–36. 17984684 10.1097/NEN.0b013e3181598294

[aww249-B25] Koike H Iijima M Sugiura M Mori K Hattori N Ito H Alcoholic neuropathy is clinicopathologically distinct from thiamine-deficiency neuropathy. Ann Neurol 2003; 54: 19–29. 12838517 10.1002/ana.10550

[aww249-B26] Kulikova-Schupak R Knupp KG Pascual JM Chin SS Kairam R Patterson MC Rectal biopsy in the diagnosis of neuronal intranuclear hyaline inclusion disease. J Child Neurol 2004; 19: 59–62. 15032387 10.1177/08830738040190010707

[aww249-B27] Kuusisto E Kauppinen T Alafuzoff I Use of p62/SQSTM1 antibodies for neuropathological diagnosis. Neuropathol Appl Neurobiol 2008; 34: 169–80. 17961133 10.1111/j.1365-2990.2007.00884.x

[aww249-B28] Langford LA Demyelinative process associated with atypical intranuclear glial inclusions. Ultrastruct Pathol 1994; 18: 15–8. 8191622 10.3109/01913129409016268

[aww249-B29] Leestma JE Andrews JM The fine structure of the Marinesco body. Arch Pathol 1969; 88: 431–6. 4898280

[aww249-B30] Lindenberg R Rubinstein LJ Herman MM Haydon GB A light and electron microscopy study of an unusual widespread nuclear inclusion body disease. A possible residuum of an old herpesvirus infection. Acta Neuropathol (Berl) 1968; 10: 54–73. 4295804 10.1007/BF00690510

[aww249-B31] Liu Y Mimuro M Yoshida M Hashizume Y Niwa H Miyao S Inclusion-positive cell types in adult-onset intranuclear inclusion body disease: implications for clinical diagnosis. Acta Neuropathol 2008; 116: 615–23. 18923837 10.1007/s00401-008-0442-7

[aww249-B32] Maddalena A Richards CS McGinniss MJ Brothman A Desnick RJ Grier RE Technical standards and guidelines for fragile X: the first of a series of disease-specific supplements to the Standards and Guidelines for Clinical Genetics Laboratories of the American College of Medical Genetics. Quality Assurance Subcommittee of the Laboratory Practice Committee. Genet Med 2001; 3: 200–5. 11388762 10.1097/00125817-200105000-00010 PMC3110344

[aww249-B33] Martin JB Banker BQ Subacute multifocal leukoencephalopathy with widespread intranuclear inclusions. Arch Neurol 1969; 21: 590–602. 4901419 10.1001/archneur.1969.00480180046003

[aww249-B34] Michaud J Gilbert JJ Multiple system atrophy with neuronal intranuclear hyaline inclusions. Report of a new case with light and electron microscopic studies. Acta Neuropathol (Berl) 1981; 54: 113–9. 6264727 10.1007/BF00689403

[aww249-B35] Mizushima T Kato M Iwanaga I Sato F Kubo K Ehira N Technical difficulty according to location, and risk factors for perforation, in endoscopic submucosal dissection of colorectal tumors. Surg Endosc 2015; 29: 133–9. 24993172 10.1007/s00464-014-3665-9

[aww249-B36] Munoz-Garcia D Ludwin SK Adult-onset neuronal intranuclear hyaline inclusion disease. Neurology 1986; 36: 785–90. 3010181 10.1212/wnl.36.6.785

[aww249-B37] O'Sullivan JD Hanagasi HA Daniel SE Tidswell P Davies SW Lees AJ Neuronal intranuclear inclusion disease and juvenile parkinsonism. Mov Disord 2000; 15: 990–5. 11009211 10.1002/1531-8257(200009)15:5<990::aid-mds1035>3.0.co;2-i

[aww249-B38] Odagiri S Tanji K Mori F Kakita A Takahashi H Kamitani T Immunohistochemical analysis of Marinesco bodies, using antibodies against proteins implicated in the ubiquitin-proteasome system, autophagy and aggresome formation. Neuropathology 2012; 32: 261–6. 22118216 10.1111/j.1440-1789.2011.01267.x

[aww249-B39] Okamoto K Hirai S Fine structures of Marinesco body and nuclear body in the substantia nigra (author's transl) [in Japanese]. Rinsho Shinkeigaku 1981; 21: 781–9. 6276067

[aww249-B40] Oyer CE Cortez S O'Shea P Popovic M Cardiomyopathy and myocyte intranuclear inclusions in neuronal intranuclear inclusion disease: a case report. Hum Pathol 1991; 22: 722–4. 1649120 10.1016/0046-8177(91)90296-2

[aww249-B41] Patel H Norman MG Perry TL Berry KE Multiple system atrophy with neuronal intranuclear hyaline inclusions. Report of a case and review of the literature. J Neurol Sci 1985; 67: 57–65. 2580060 10.1016/0022-510x(85)90022-x

[aww249-B42] Paviour DC Revesz T Holton JL Evans A Olsson JE Lees AJ Neuronal intranuclear inclusion disease: report on a case originally diagnosed as dopa-responsive dystonia with Lewy bodies. Mov Disord 2005; 20: 1345–9. 15966005 10.1002/mds.20559

[aww249-B43] Prins ND van Dijk EJ den Heijer T Vermeer SE Jolles J Koudstaal PJ Cerebral small-vessel disease and decline in information processing speed, executive function and memory. Brain 2005; 128 (Pt 9): 2034–41. 15947059 10.1093/brain/awh553

[aww249-B44] Prust M Wang J Morizono H Messing A Brenner M Gordon E GFAP mutations, age at onset, and clinical subtypes in Alexander disease. Neurology 2011; 77: 1287–94. 21917775 10.1212/WNL.0b013e3182309f72 PMC3179649

[aww249-B45] Sasaki T Hideyama T Saito Y Shimizu J Maekawa R Shiio Y Neuronal intranuclear inclusion disease presenting with recurrent cerebral infarct-like lesions. Neurol Clin Neurosci 2015; 3: 185–7.

[aww249-B46] Sawaishi Y Review of Alexander disease: beyond the classical concept of leukodystrophy. Brain Dev 2009; 31: 493–8. 19386454 10.1016/j.braindev.2009.03.006

[aww249-B47] Schmahmann JD Smith EE Eichler FS Filley CM Cerebral white matter: neuroanatomy, clinical neurology, and neurobehavioral correlates. Ann N Y Acad Sci 2008; 1142: 266–309. 18990132 10.1196/annals.1444.017 PMC3753195

[aww249-B48] Schuffler MD Bird TD Sumi SM Cook A A familial neuronal disease presenting as intestinal pseudoobstruction. Gastroenterology 1978; 75: 889–98. 212342

[aww249-B49] Sloane AE Becker LE Ang LC Wark J Haslam RH Neuronal intranuclear hyaline inclusion disease with progressive cerebellar ataxia. Pediatr Neurol 1994; 10: 61–6. 7515242 10.1016/0887-8994(94)90070-1

[aww249-B50] Sone J Hishikawa N Koike H Hattori N Hirayama M Nagamatsu M Neuronal intranuclear hyaline inclusion disease showing motor-sensory and autonomic neuropathy. Neurology 2005; 65: 1538–43. 16301479 10.1212/01.wnl.0000184490.22527.90

[aww249-B51] Sone J Kitagawa N Sugawara E Iguchi M Nakamura R Koike H Neuronal intranuclear inclusion disease cases with leukoencephalopathy diagnosed via skin biopsy. J Neurol Neurosurg Psychiatry 2014; 85: 354–6. 24039026 10.1136/jnnp-2013-306084

[aww249-B52] Sone J Tanaka F Koike H Inukai A Katsuno M Yoshida M Skin biopsy is useful for the antemortem diagnosis of neuronal intranuclear inclusion disease. Neurology 2011; 76: 1372–6. 21411744 10.1212/WNL.0b013e3182166e13

[aww249-B53] Sung JH Light, fluorescence, and electron microscopic features of neuronal intranuclear hyaline inclusions associated with multisystem atrophy. Acta Neuropathol 1980; 50: 115–20. 6249061 10.1007/BF00692861

[aww249-B54] Sung JH Ramirez-Lassepas M Mastri AR Larkin SM An unusual degenerative disorder of neurons associated with a novel intranuclear hyaline inclusion (neuronal intranuclear hyaline inclusion disease). A clinicopathological study of a case. J Neuropathol Exp Neurol 1980; 39: 107–30. 6154779 10.1097/00005072-198003000-00001

[aww249-B55] Takahashi-Fujigasaki J Neuronal intranuclear hyaline inclusion disease. Neuropathology 2003; 23: 351–9. 14719553 10.1046/j.1440-1789.2003.00524.x

[aww249-B56] Toyota T Huang Z Nohara S Okada K Kakeda S Korogi Y Neuronal intranuclear inclusion disease manifesting with new-onset epilepsy in the elderly. Neurol Clin Neurosci 2015; 3: 238–40.

[aww249-B57] Weidenheim KM Dickson DW Intranuclear inclusion bodies in an elderly demented woman: a form of intranuclear inclusion body disease. Clin Neuropathol 1995; 14: 93–9. 7606903

[aww249-B58] Wiltshire KM Dunham C Reid S Auer RN Suchowersky O Neuronal Intranuclear Inclusion Disease presenting as juvenile Parkinsonism. Can J Neurol Sci 2010; 37: 213–8. 20437931 10.1017/s031716710000994x

[aww249-B59] Woulfe JM Abnormalities of the nucleus and nuclear inclusions in neurodegenerative disease: a work in progress. Neuropathol Appl Neurobiol 2007; 33: 2–42. 17239006 10.1111/j.1365-2990.2006.00819.x

[aww249-B60] Zannolli R Gilman S Rossi S Volpi N Bernini A Galluzzi P Hereditary neuronal intranuclear inclusion disease with autonomic failure and cerebellar degeneration. Arch Neurol 2002; 59: 1319–26. 12164731 10.1001/archneur.59.8.1319

