# Supplementary material for: Clinicopathological features of adult-onset neuronal intranuclear inclusion disease
Source: Brain. 2016 Oct 25;139(12):3170–86. doi: 10.1093/brain/aww249 (PMC5382941; doi:10.1093/brain/aww249)
Supplement: Supplementary Data [file aww249_supp.zip › brain-2016-00640-File012.pdf]

Supplemental Table 3 Laboratory data of each adult onset NIID cases

	Family Patient	Age/ Sex	CK (U/l)	CSF			HgbA1c (NGSP) (%)	Nerve conduction				*FMR1* premutation
								Motor		Sensory		
				Cell (/μl)	Protein (mg/dl)	Glucose (mg/dl)		MCV slowing	CMAP reduction	SCV slowing	SNAP reduction	
	S-1	61F	120	2	40	75	5.7	+	-	-	-	-
	S-2	62M	94	12	70	68	6.0	+	-	-	-	-
	S-3	66M	n.a.	n.a.	n.a.	n.a.	5.7	+	-	+	-	-
	S-4	67F	78	1	48	84	7.0	n.a.	n.a.	n.a.	n.a.	-
	S-5	67F	222	2	30	77	n.a.	n.a.	n.a.	n.a.	n.a.	n.a.
	S-6	75F	92	1	35	68	6.4	+	-	+	-	-
	S-7	70F	60	n.a.	n.a.	n.a.	5.5	+	-	+	-	n.a.
	S-8	73F	87	1	58	75	n.a.	+	-	+	+	-
	S-9	57M	113	1	66	74	5.5	+	-	+	-	-
	S-10	63M	33	n.a.	n.a.	n.a.	6.9	+	-	+	-	-
	S-11	68F	124	3	77	67	5.9	+	-	-	+	-
	S-12	73F	n.a.	4	58	63	5.8	n.a.	n.a.	n.a.	n.a.	-
	S-13	73M	55	0	43	78	5.6	+	-	+	-	-
	S-14	78M	81	6	282	67	5.6	+	+	+	-	n.a.
	S-15	66F	59	2	70	68	6.3	+	-	+	-	n.a.
	S-16	69F	n.a.	0	30	60	n.a.	-	-	-	-	-
	S-17	69F	90	n.a.	n.a.	n.a.	6.2	+	-	+	-	-
	S-18	56M	274	4	101	73	n.a.	+	+	+	-	n.a.
Sporadic cases	S-19	62M	n.a.	n.a.	n.a.	n.a.	7.1	n.a.	n.a.	n.a.	n.a.	-
	S-20	70M	67	8	53	59	5.5	+	-	+	+	-
	S-21	66M	66	2	31	69	5.5	+	-	-	-	n.a.
	S-22	69M	69	n.a.	n.a.	n.a.	6.6	n.a.	n.a.	n.a.	n.a.	-
	S-23	70F	61	9	27	78	6.1	+	+	+	-	-
	S-24	74M	114	4	76	58	5.6	+	+	+	-	-
	S-25	67F	419	1	42.5	69	5.3	+	-	-	-	-
	S-26	70F	73	1	72	68	5.8	+	-	+	-	-
	S-27	71F	33	n.a.	n.a.	n.a.	5.1	+	+	-	-	-
	S-28	74F	149	1	54	66	5.9	+	-	+	+	-
	S-29	70M	176	9	66	79	5.4	+	-	+	-	-
	S-30	74F	96	n.a.	n.a.	n.a.	5.8	+	-	+	-	n.a.
	S-31	75F	42	2	114	108	7.7	n.a.	n.a.	n.a.	n.a.	-
	S-32	81F	35	1	54	68	5.7	+	-	+	-	n.a.
	S-33	61F	18	n.a.	n.a.	n.a.	5.4	+	+	+	-	n.a.
	S-34	65F	70	4	35	61	5.3	+	-	-	-	n.a.
	S-35	74F	n.a.	3	144	72	n.a.	n.a.	n.a.	n.a.	n.a.	-
	S-36	75F	86	2	37	55	5.3	+	-	+	-	-
	S-37	68F	61	2	60	63	5.4	+	-	+	-	n.a.
	S-38	71F	33	4	66	64	5.5	+	-	+	-	-
Incidence rate			9.1%	17.2%	65.5%	0%	21.2%	96.7%.	19.4%	74.2%.	12.9%	0%
	F1-1	67M	35	1	65	86	5.9	+	-	+	-	-
	F1-2	59M	298	n.a.	n.a.	n.a.	6.9	+	+	n.e	n.e	-
	F1-3	53F	356	n.a.	n.a.	n.a.	5.7	+	+	+	+	-
	F1-4	48M	411	n.a.	n.a.	n.a.	8.0	+	+	+	-	-
	F1-5	36F	359	n.a.	n.a.	n.a.	n.a.	n.a.	n.a.	n.a.	n.a.	n.a.
	F1-6	34M	n.a.	n.a.	n.a.	n.a.	n.a.	n.a.	n.a.	n.a.	n.a.	n.a.
	F1-7	37F	n.a.	n.a.	n.a.	n.a.	n.a.	n.a.	n.a.	n.a.	n.a.	n.a.
	F1-8	35M	559	n.a.	n.a.	n.a.	n.a.	+	-	+	+	-
Familial cases	F2-1	48F	n.a.	n.a.	n.a.	n.a.	n.a.	+	n.a.	+	n.a.	n.a.
	F2-2	45F	276	3	40	67	5.4	+	+	+	+	-
	F3-1	64F	302	W.N.L.	W.N.L.	W.N.L.	n.a.	+	+	+	+	-
	F4-1	71F	95	2	58	61	5.5	-	-	+	-	n.a.
	F4-2	59F	225	1	43	60	n.a.	+	-	-	-	-
	F4-3	57F	29	1	34	n.a.	n.a.	-	-	-	-	n.a.
	F4-4	56F	66	1	26	n.a.	5.2	n.a.	n.a.	n.a.	n.a.	n.a.
	F5-1	57M	120	3	184	67	5.1	n.a.	n.a.	n.a.	n.a.	-
	F5-2	59F	83	1	29	69	5.7	+	-	+	+	n.a.
	F6-1	76F	n.a.	n.a.	n.a.	n.a.	n.a.	n.a.	n.a.	n.a.	n.a.	n.a.
	F6-2	68F	n.a.	3	59	80	n.a.	n.a.	n.a.	n.a.	n.a.	n.a.
Incidence rate			57.1%	0%	40.0%	0%	22.2%	83.3%	45.5%	83.3%	54.5%	0%

n.a = not available: n.e = not evoked, W.N.L. = within normal limit; CK=creatine kinase; NGSP=national glycohemoglobin standardization program, MCV=motor nerve conduction velocity; CMAP=compound muscle action potential; SCV= sensory nerve conduction velocity; SNAP=sensory nerve action potential; CMAP reduction and SNAP reduction were determined that each value below control average value -2SD (Koike et al, 2003).
