# Supplementary material for: Clinicopathological features of adult-onset neuronal intranuclear inclusion disease
Source: Brain. 2016 Oct 25;139(12):3170–86. doi: 10.1093/brain/aww249 (PMC5382941; doi:10.1093/brain/aww249)
Supplement: Supplementary Data [file aww249_supp.zip › brain-2016-00640-File010.pdf]

Supplemental Table 1 Clinical manifestations of each adult onset NIID cases - 1

	Family Patient	Diagnose	Age/ Sex	Onset age (y)	Duration (y)	Weak-ness	Sensory disturb-ance	Autonomic				Dementia	Tremor	Rigidity	Ataxia
								Vomiting	Bladder dysfunc-tion	Syncope	Miosis				
Sporadic cases	S-1	skin biopsy	61F	**61**	**1**	-	+	-	n.a.	-	n.a.	+	-	-	+
	S-2	skin biopsy	62M	**62**	**1**	-	-	-	-	-	+	+	-	-	+
	S-3	skin biopsy	66M	**66**	**1**	-	-	-	-	-	n.a.	+	+	-	+
	S-4	skin biopsy	67F	**67**	**1**	-	-	-	-	-	+	+	-	-	-
	S-5	section	67F	**67**	**1**	-	-	-	n.a.	n.a.	n.a.	+	-	-	-
	S-6	skin biopsy	75F	**75**	**1**	-	+	-	+	-	+	+	-	-	+
	S-7	skin biopsy	70F	**69**	**2**	-	-	-	-	-	+	+	-	-	-
	S-8	skin biopsy	73F	**72**	**2**	+	-	-	-	-	n.a.	+	-	-	+
	S-9	skin biopsy	57M	**55**	**3**	+	+	-	-	-	+	+	-	-	-
	S-10	skin biopsy	63M	**61**	**3**	-	-	-	-	-	+	+	-	-	-
	S-11	skin biopsy	68F	**66**	**3**	-	-	-	-	-	n.a.	+	-	-	-
	S-12	skin biopsy	73M	**71**	**3**	-	-	-	+	-	+	+	-	-	+
	S-13	skin biopsy	73F	**71**	**3**	-	-	-	+	-	-	-	+	-	-
	S-14	skin biopsy	78M	**76**	**3**	-	+	-	-	-	+	+	-	-	+
	S-15	skin biopsy	66F	**63**	**4**	+	-	-	+	-	+	+	-	-	+
	S-16	skin biopsy	69F	**66**	**4**	-	-	-	-	+	n.a.	+	-	-	+
	S-17	skin biopsy	69F	**66**	**4**	-	-	-	-	-	n.a.	+	-	-	+
	S-18	skin biopsy	56M	**52**	**5**	-	+	-	-	-	n.a.	+	-	-	+
	S-19	skin biopsy	62M	**57**	**6**	+	+	-	-	-	n.a.	+	-	-	-
	S-20	skin biopsy	70M	**65**	**6**	-	-	-	-	-	n.a.	+	-	-	-
	S-21	skin biopsy	66M	**60**	**7**	-	+	-	+	-	n.a.	+	-	-	+
	S-22	skin biopsy	69M	**63**	**7**	-	-	-	+	-	n.a.	+	-	+	-
	S-23	skin biopsy	70F	**64**	**7**	+	-	+	-	-	n.a.	+	+	+	-
	S-24	skin biopsy	74M	**67**	**8**	-	-	-	+	-	+	+	-	-	+
	S-25	skin biopsy	67F	**59**	**9**	-	-	+	-	-	n.a.	+	-	-	-
	S-26	skin biopsy	70F	**62**	**9**	-	+	-	+	-	+	+	-	-	+
	S-27	skin biopsy	71F	**64**	**9**	+	n.a.	-	+	-	+	+	-	-	-
	S-28	skin biopsy	74F	**66**	**9**	+	-	+	n.a.	-	+	+	-	-	-
	S-29	skin biopsy	70M	**61**	**10**	+	+	-	+	-	+	+	-	-	+
	S-30	skin biopsy	74F	**63**	**10**	-	-	-	+	+	+	+	+	+	-
	S-31	B / S	75F	**66**	**10**	n.a.	n.a.	-	+	-	+	+	-	n.a.	n.a.
	S-32	skin biopsy	81F	**72**	**10**	+	-	+	n.a.	+	n.a.	+	+	+	+
	S-33	skin biopsy	61F	**51**	**11**	+	n.a.	-	-	-	n.a.	+	+	+	n.a.
	S-34	skin biopsy	65F	**55**	**11**	-	-	-	-	-	+	+	-	-	+
	S-35	skin biopsy	74F	**64**	**11**	-	+	-	-	-	n.a.	+	+	-	-
	S-36	skin biopsy	75F	**65**	**11**	-	-	-	-	-	n.a.	+	-	-	+
	S-37	skin biopsy	68F	**55**	**14**	-	-	-	-	-	n.a.	+	+	+	+
	S-38	skin biopsy	71F	**53**	**19**	-	-	-	-	-	n.a.	+	-	-	-
	Incidence rate					**27.0%**	**28.6%**	**10.5%**	**35.3%**	**8.1%**	**94.4%**	**97.4%**	**21.1%**	**16.2%**	**52.8%**
Familial cases	F1-1	Section	67M	**30**	**38**	++	+	+	+	-	+	-	-	-	-
	F1-2	Section	59M	**16**	**44**	++	++	+	+	-	+	-	-	-	-
	F1-3	skin biopsy	53F	**30**	**24**	+	+	+	+	-	n.a.	-	-	-	-
	F1-4	skin biopsy	48M	**20**	**28**	+	+	-	+	-	-	-	-	-	-
	F1-5	skin biopsy	36F	**30**	**7**	+	-	-	-	-	n.a.	-	-	-	-
	F1-6	skin biopsy	34M	**26**	**9**	+	-	-	-	-	n.a.	-	-	-	-
	F1-7	skin biopsy	37F	**30**	**8**	+	+	-	+	-	n.a.	-	-	-	-
	F1-8	skin biopsy	35M	**33**	**3**	+	+	-	-	-	-	-	-	-	-
	F2-1	Section	48F	**26**	**23**	++	+	+	+	-	n.a.	-	-	-	-
	F2-2	skin biopsy	45F	**23**	**22**	+	+	-	-	-	n.a.	-	-	-	-
	F3-1	skin biopsy	64F	**39**	**26**	++	+	+	+	-	+	+	-	-	-
	F4-1	skin biopsy	71F	**68**	**4**	+	+	+	+	-	+	+	+	-	-
	F4-2	skin biopsy	59F	**54**	**6**	+	+	-	+	-	+	+	+	-	-
	F4-3	skin biopsy	57F	**54**	**4**	+	-	-	-	-	+	+	-	+	-
	F4-4	skin biopsy	56F	**56**	**1**	+	+	-	-	-	-	+	-	-	-
	F5-1	skin biopsy	57M	**43**	**15**	+	n.a.	-	n.a.	-	+	+	-	n.a.	n.a.
	F5-2	skin biopsy	59F	**47**	**13**	+	+	-	+	-	-	+	-	-	+
	F6-1	skin biopsy	76F	**66**	**11**	-	-	-	n.a.	-	n.a.	+	+	+	-
	F6-2	n.a.	68F	**62**	**7**	+	-	-	n.a.	-	n.a.	+	-	+	-
	Incidence rate					**94.7%**	**72.2%**	**31.6%**	**62.5%**	**0%**	**63.6%**	**47.3%**	**15.8%**	**16.7%**	**5.6%**

n.a = not available; B/S = skin biopsy and section.
