# Supplementary material for: Clinicopathological features of adult-onset neuronal intranuclear inclusion disease
Source: Brain. 2016 Oct 25;139(12):3170–86. doi: 10.1093/brain/aww249 (PMC5382941; doi:10.1093/brain/aww249)
Supplement: Supplementary Data [file aww249_supp.zip › brain-2016-00640-File011.pdf]

Supplemental Table 2 Clinical manifestations of each adult onset NIID cases - 2

	Family Patient	Age/ Sex	Dementia	Abnormal behavior	Generalized convulsion	Disturbance of consciousness	Encephalitic episode	MRI			SPECT	Executive function tests	
								Leukoencep halopathy	DWI U-fiber high	Ventricular distention	hypo perfusion	MMSE	FAB
Sporadic cases	S-1	61F	+	-	-	-	-	-	+	-	n.a.	26	n.a.
	S-2	62M	+	-	-	-	-	+	+	+	+	30	17
	S-3	66M	+	-	-	-	-	+	+	+	+	27	n.a.
	S-4	67F	+	+	-	+	-	+	+	+	+	29	n.a.
	S-5	67F	+	-	-	+	+	+	+	+	n.a.	5	n.a.
	S-6	75F	+	-	+	-	-	+	+	+	n.a.	25	n.a.
	S-7	70F	+	-	-	-	-	+	+	+	n.a.	19	6
	S-8	73F	+	-	-	-	-	+	+	+	+	25	15
	S-9	57M	+	-	+	+	+	+	+	+	+	15	6
	S-10	63M	+	-	+	-	+	+	+	+	n.a.	12	n.a.
	S-11	68F	+	-	-	+	-	+	+	+	+	19	9
	S-12	73M	+	-	-	-	-	+	+	+	-	23	14
	S-13	73F	-	-	-	-	-	+	+	+	+	26	14
	S-14	78M	+	+	-	-	-	+	+	+	n.a.	26	11
	S-15	66F	+	-	-	-	-	+	+	+	+	25	n.a.
	S-16	69F	+	-	-	-	-	+	+	+	+	29	n.a.
	S-17	69F	+	-	-	-	-	+	+	+	n.a.	21	n.a.
	S-18	56M	+	-	-	+	-	+	+	+	n.a.	26	13
	S-19	62M	+	-	-	-	-	+	+	+	n.a.	13	10
	S-20	70M	+	+	-	-	-	+	+	+	+	n.a.	n.a.
	S-21	66M	+	-	-	+	-	+	+	+	+	22	10
	S-22	69M	+	-	-	+	-	+	+	+	+	22	10
	S-23	70F	+	-	-	+	+	+	+	+	n.a.	n.a.	n.a.
	S-24	74M	+	-	-	-	-	+	+	+	+	21	7
	S-25	67F	+	+	-	+	+	+	+	+	+	n.a.	n.a.
	S-26	70F	+	-	-	-	-	+	+	+	+	27	12
	S-27	71F	+	+	-	-	-	+	+	+	+	0	n.a.
	S-28	74F	+	+	-	+	-	+	+	+	+	n.a.	n.a.
	S-29	70M	+	-	-	-	-	+	+	+	n.a.	28	n.a.
	S-30	74F	+	-	-	+	-	+	+	+	n.a.	15	n.a.
	S-31	75F	+	-	-	+	-	+	+	+	n.a.	20	n.a.
	S-32	81F	+	-	-	-	-	+	+	+	+	15	n.a.
	S-33	61F	+	+	+	+	-	+	+	+	+	n.a.	n.a.
	S-34	65F	+	+	-	-	-	+	+	+	+	26	9
	S-35	74F	+	-	-	-	+	+	+	+	n.a.	30	12
	S-36	75F	+	+	+	+	+	+	+	+	+	n.a.	n.a.
	S-37	68F	+	+	-	-	-	+	+	+	+	16	7
	S-38	71F	+	-	-	+	+	+	+	+	n.a.	29	13
	Incidence rate		97.4%	26.3%	13.2%	39.5%	21.0%	97.4%	100%	97.4%	95.7%	50%	94.4%
Familial cases	F1-1	67M	-	-	-	-	-	+	n.a.	+	n.a.	n.a.	n.a.
	F1-2	59M	-	-	-	-	-	n.a.	n.a.	n.a.	n.a.	n.a.	n.a.
	F1-3	53F	-	-	-	-	-	-	n.a.	-	n.a.	n.a.	n.a.
	F1-4	48M	-	-	-	-	-	-	-	-	n.a.	26	16
	F1-5	36F	-	-	-	-	-	n.a.	n.a.	n.a.	n.a.	n.a.	n.a.
	F1-6	34M	-	-	-	-	-	n.a.	n.a.	n.a.	n.a.	n.a.	n.a.
	F1-7	37F	-	-	-	-	-	n.a.	n.a.	n.a.	n.a.	n.a.	n.a.
	F1-8	35M	-	-	-	-	-	n.a.	n.a.	n.a.	n.a.	n.a.	n.a.
	F2-1	48F	-	-	-	-	-	n.a.	n.a.	n.a.	n.a.	n.a.	n.a.
	F2-2	45F	-	-	-	-	-	-	-	-	+	28	17
	F3-1	64F	+	-	-	+	-	+	+	+	+	25	n.a.
	F4-1	71F	+	-	-	+	-	+	+	+	n.a.	26	n.a.
	F4-2	59F	+	-	-	-	-	+	+	+	n.a.	26	n.a.
	F4-3	57F	+	-	+	+	-	+	+	+	n.a.	n.a.	n.a.
	F4-4	56F	+	-	-	-	-	+	+	+	n.a.	n.a.	n.a.
	F5-1	57M	+	+	-	+	+	+	+	+	+	n.a.	n.a.
	F5-2	59F	+	-	-	-	-	+	+	+	-	28	14
	F6-1	76F	+	-	-	-	-	+	+	+	+	25	n.a.
	F6-2	68F	+	+	-	+	-	+	+	+	n.a.	25	n.a.
	Incidence rate		47.4%	10.5%	5.3%	26.3%	5.3%	76.9%	81.8%	76.9%	80%	0%	100%

n.a = not available; MMSE=mini mental state examination, FAB=frontal assessment battery, We adopted 24 as the cut off score for MMSE as previous reports (Folstein et al., 1975; Diniz et al., 2007), and published age-matched average as cut off score for FAB (Dubois et al., 2000; Appollonio et al., 2005),
